# Long-term nitrogen deposition enhances microbial capacities in soil carbon stabilization but reduces network complexity

**DOI:** 10.1186/s40168-022-01309-9

**Published:** 2022-07-28

**Authors:** Xingyu Ma, Tengxu Wang, Zhou Shi, Nona R. Chiariello, Kathryn Docherty, Christopher B. Field, Jessica Gutknecht, Qun Gao, Yunfu Gu, Xue Guo, Bruce A. Hungate, Jiesi Lei, Audrey Niboyet, Xavier Le Roux, Mengting Yuan, Tong Yuan, Jizhong Zhou, Yunfeng Yang

**Affiliations:** 1 State Key Joint Laboratory of Environment Simulation and Pollution Control, School of Environment, Tsinghua University, Beijing, 100084 China; 2 China Urban Construction Design & Research Institute Co., Ltd, Beijing, 100120 China; 3 North China Municipal Engineering Design & Research Institute Co., Ltd., the Beijing Branch, Beijing, 100081 China; 4 Institute for Environmental Genomics and Department of Microbiology and Plant Biology, University of Oklahoma, Norman, OK 73019 USA; 5 Department of Global Ecology, Carnegie Institution for Science, Stanford, CA 94305 USA; 6 Department of Biological Sciences, Western Michigan University, Kalamazoo, MI 49008 USA; 7 Department of Soil Ecology, Helmholtz Centre for Environmental Research – UFZ, 06120 Halle, Germany; 8 Present address: Department of Soil, Water, and Climate, University of Minnesota, Twin Cities, Saint Paul, MN 55104 USA; 9 Department of Microbiology, College of Resource, Sichuan Agricultural University, Chengdu, 611130 China; 10 Center for Ecosystem Science and Society, and Department of Biological Sciences, Northern Arizona University, Flagstaff, AZ 86011 USA; 11 Sorbonne Université, Université Paris Cité, UPEC, CNRS, INRAE, IRD, Institut d’Ecologie et des Sciences de l’Environnement de Paris, iEES-Paris, Paris, France; 12 AgroParisTech, Paris, France; 13 Microbial Ecology Centre LEM, INRAE, CNRS, University of Lyon, University Lyon 1, VetAgroSup, UMR INRAE 1418, 43 boulevard du 11 novembre 1918, 69622 Villeurbanne, France; 14 Department of Environmental Science, Policy and Management, University of California Berkeley, Berkeley, CA 94720 USA; 15 Earth Sciences Division, Lawrence Berkeley National Laboratory, Berkeley, CA 94720 USA

**Keywords:** Soil microbial community, Nitrogen deposition, High-throughput sequencing, GeoChip, Global change

## Abstract

**Background:**

Anthropogenic activities have increased the inputs of atmospheric reactive nitrogen (N) into terrestrial ecosystems, affecting soil carbon stability and microbial communities. Previous studies have primarily examined the effects of nitrogen deposition on microbial taxonomy, enzymatic activities, and functional processes. Here, we examined various functional traits of soil microbial communities and how these traits are interrelated in a Mediterranean-type grassland administrated with 14 years of 7 g m^−2^ year^−1^ of N amendment, based on estimated atmospheric N deposition in areas within California, USA, by the end of the twenty-first century.

**Results:**

Soil microbial communities were significantly altered by N deposition. Consistent with higher aboveground plant biomass and litter, fast-growing bacteria, assessed by abundance-weighted average rRNA operon copy number, were favored in N deposited soils. The relative abundances of genes associated with labile carbon (C) degradation (e.g., *amyA* and *cda*) were also increased. In contrast, the relative abundances of functional genes associated with the degradation of more recalcitrant C (e.g., *mannanase* and *chitinase*) were either unchanged or decreased. Compared with the ambient control, N deposition significantly reduced network complexity, such as average degree and connectedness. The network for N deposited samples contained only genes associated with C degradation, suggesting that C degradation genes became more intensely connected under N deposition.

**Conclusions:**

We propose a conceptual model to summarize the mechanisms of how changes in above- and belowground ecosystems by long-term N deposition collectively lead to more soil C accumulation.

Video Abstract

**Supplementary Information:**

The online version contains supplementary material available at 10.1186/s40168-022-01309-9.

## Background

Anthropogenic activities have dramatically increased the production of reactive N worldwide, likely at levels exceeding all of the natural terrestrial N sources combined [1]. Increased N inputs into the environment have many consequences, including marine and freshwater eutrophication, marine acidification, air pollution, and changes in ecosystem functioning and composition [2, 3]. Several studies have shown that soil microbial taxonomic diversity is either unaltered or decreased with N deposition, often with a higher relative abundance of *Proteobacteria* and a lower relative abundance in *Acidobacteria* [4–7]. N depositions, especially those at high levels (> 50 kg ha^−1^ year^−1^ of N) or over long durations, often decrease microbial biomass [8, 9] and soil respiration [10]. However, the activities of hydrolases can increase with N deposition, showing preferential degradation of labile C pools [11, 12]. By contrast, recalcitrant C-degrading enzymes such as phenol oxidases remain unchanged [5, 11] or decrease [13, 14], suggesting that an increase in labile C pools might ameliorate microbial requirements for obtaining N from recalcitrant C pools, which are richer in the N content than labile C [15]. Additionally, N deposition often increases N cycling rates, as reported for gross N mineralization, gross and potential nitrification or ammonia and nitrite oxidation, and potential denitrification [16–18]. N deposition can also alter the abundances of key microbial groups involved in N cycling, stimulating some bacterial but not archaeal ammonia oxidizers and increasing the abundance of some nitrite oxidizers such as *Nitrobacter* but not *Nitrospira* [19–23]. However, N deposition can increase, decrease, or do not change the abundances of N fixation groups in different environments such as cold region soils, forest soils, and rhizosphere [24–26].

Plant growth is generally N limited in annual grasslands of California, USA [27]. Thus, N deposition typically induces large changes in plant and soil microbial community composition [7, 28–30]. Although many studies have examined the responses of below- and aboveground ecosystems to N addition, much remains unknown how responses of a broad range of microbial functional groups and interactions are related to soil C and N dynamics, which restricts our understanding of the effect of long-term N deposition on soil microbial communities and soil functioning in natural grasslands. To address it, we carried out a 14-year N (in the form of Ca(NO_3_)_2_) deposition experiment in a multifactor field study in a California annual grassland located near Stanford, CA, USA (Jasper Ridge Global Change Experiment, JRGCE) [31], which is one of the longest experiments of N deposition. The JRGCE is designed to assess grassland responses to single and multiple drivers of global changes, including elevated CO_2_, warming, atmospheric nitrate deposition, and enhanced precipitation. Each experimental plot at the JRGCE is circular, 2 m in diameter, and equally divided into four quadrants of 0.78 m^2^ [31]. The CO_2_ and warming treatments are applied at the plot level, and N and water treatments are applied at the quadrant level in a full factorial design, resulting in 16 treatments with eight replicates of each. However, an experimental fire burned four replicate blocks in 2011. Therefore, only the four unburned blocks (a total of 64 samples) are used in this study.

We used 16S rRNA gene amplicon sequencing (Illumina MiSeq) and functional gene array (GeoChip 4.6) to compare the microbial community compositions (both taxonomic diversity and functional diversity) between control and N deposited samples. Our objectives are to assess the long-term effect of N deposition on a broad range of soil bacterial taxa, major functional genes associated with C and N cycling, and how these genes are co-occurring. We also aim to analyze how the changes in the taxonomic and functional gene compositions are related to the changes in environmental attributes. Since N deposition typically stimulates the primary productivity of plants and consequently fresh C input to soil [8, 32, 33], we hypothesize that fast-growing bacteria would be enriched and that functional genes associated with labile C degradation would be stimulated by N deposition, leading to higher metabolic capacities for soil C stabilization. Accordingly, we hypothesize that N deposition will affect associations among functional groups as characterized by network analysis. Since previous results at our study site have shown that soil ammonium concentrations increase with Ca(NO_3_)_2_ deposition over the long term owing to N turnover and enhanced N mineralization [17, 22, 34], our third hypothesis is that functional microbial groups associated with N cycling would be stimulated. More precisely, we postulate that microbial groups that perform better under higher N availability, e.g., bacterial ammonia oxidizers and *Nitrobacter*-like nitrite oxidizers [19–21], would be particularly stimulated.

## Materials and methods

### Site and soil description

The experiment was conducted at the Jasper Ridge Global Change Experiment (JRGCE) site in coastal central California, USA. It has been operated on an annual grassland under a Mediterranean-type climate at the Jasper Ridge Biological Preserve (37° 24′ N, 122° 13′ W) beginning in October 1998. The plant community comprises annual and perennial grasses and forbs [29]. The soil is a fine Haploxeralf developed from Franciscan complex alluvium sandstone.

Soil samples were collected in April 2012, i.e., 14 years after the experiment began. On April 26 and 27, 2012, we collected soil cores (5 cm diameter × 7 cm deep) to generate each of 32 control samples and 32 long-term N deposited samples. Soil samples were thoroughly mixed and sieved through a 2-mm mesh to remove visible roots and rocks and stored at −80 °C before DNA extraction, or at −20°C before a range of soil geochemical measurements.

### DNA extraction, purification, and quantification

DNA was extracted from 5 g of well-mixed soil using a freeze grinding mechanical lysis method as previously described [35]. DNA was then purified by agarose gel electrophoresis, followed by extractions with phenol and chloroform and precipitation with butanol. DNA quality was determined by light absorbance ratio at wavelengths of 260/280nm and 260/230nm using a Nanodrop (NanoDrop Technologies Inc., Wilmington, DE, USA). Then, DNA concentrations were quantified with PicoGreen [36] using a FLUOstar Optima microplate reader (BMG Labtech, Jena, Germany).

### 16S rRNA gene amplicon sequencing and data processing

To study the diversity of the bacterial community, we targeted the V4 hypervariable region of 16S rRNA genes. Briefly, we carried out PCR amplification using the primer pair 515F (5′-GTGCCAGCMGCCGCGGTAA-3′) and 806R (5′-GGACTACHVGGGTWTCTAAT-3′) [37] and then sequenced the PCR products by 2 × 250 bp paired-end sequencing on a MiSeq instrument (Illumina, San Diego, CA, USA). We processed raw sequence data on the Galaxy platform with several software tools [36]. First, we carried out demultiplexing to remove PhiX sequences and sorted sequences to the appropriate samples based on their barcodes, allowing for 1 or 2 mismatches. These sequences were then trimmed based on quality scores using Btrim [38], and paired-end reads were merged into full-length sequences with FLASH [39]. After removing sequences of less than 200 bp or containing ambiguous bases, we discarded chimeric sequences based on the prediction by Uchime [40] using the reference database mode. We clustered sequences with Uclust [41] at the 97% similarity level. Final OTUs were generated based on the clustering results, and taxonomic annotation of individual OTU was assigned based on representative sequences using RDP’s 16S rRNA gene classifier [42]. The rRNA operon copy number for each OTU was estimated through the rrnDB database based on its closest relative with a known rRNA operon copy number [43]. The abundance-weighted mean rRNA operon copy number of each sample was then calculated according to a previous publication [36].

### GeoChip hybridization and raw data processing

We carried out DNA hybridization with GeoChip 4.6, as previously described [44, 45]. In brief, DNA was labeled with Cy-3 fluorescent dye using a random priming method, purified, and dried at 45 °C (SpeedVac, ThermoSavant, Milford, MA, USA). DNA was hybridized with GeoChip 4.6 at 42 °C for 16 h on an MAUI hybridization station (BioMicro, Salt Lake City, UT, USA), which was scanned with a NimbleGen MS 200 Microarray Scanner (Roche, Basel, Switzerland).

As previously described [44, 45], we processed raw GeoChip data using the following steps: (i) removing the spots with a signal-to-noise ratio (SNR) less than 2.0; (ii) log-transforming the data and then, on each microarray, dividing them by the mean intensity of all the genes; and (iii) removing genes detected only once in four replicates.

### Network analyses

Using microbial functional genes associated with C and N cycling, we constructed association networks by an in-house pipeline (http://ieg2.ou.edu/MENA) for both control and long-term N deposited samples. Only genes detected in more than 24 of the 32 biological replicates were kept for network construction. We used random matrix theory (RMT) to automatically determine the similarity threshold (St) among genes before network construction since RMT can distinguish system-specific, nonrandom associations from random gene associations [46]. Similarity matrices were calculated based on Spearman rank correlation. The network topological properties, including the total number of nodes, the total number of links, the proportion of positive and negative links, average degree (avgK), centralization of degree (CD), average clustering coefficient (avgCC), harmonic geodesic distance (HD), centralization of betweenness (CB), centralization of stress centrality (CS), and network density and modularity, were computed. The networks were graphed using Gephi v. 0.92 [47].

### Measurements of vegetation and soil attributes

We collected plant aboveground biomass from a 141-cm^2^ area of each 7853 cm^2^ quadrant 1 day before soil sampling in April 2012. The biomass of individual plant species was combined into functional groups, including the biomass of annual grasses (AG), perennial grasses (PG), annual forbs (AF), and perennial forbs (AP), plus total aboveground litter mass. We estimated root biomass by separating roots from two soil cores (2.5-cm diameter by 15-cm depth) taken in the same area used for the aboveground biomass harvest.

We measured hourly soil temperature during 2012 using thermocouples installed at a depth of 2 cm. Soil moisture was measured by drying 10 g of freshly collected soil in a 105°C oven for 1 week, and soil pH using 5 g of soil dissolved in distilled water. The total soil C (TC) and N (TN) concentrations were determined by combustion analysis on a Carlo Erba Strumentazione Model 1500 Series I analyzer at the Carnegie Institution for Science, Department of Global Ecology. To measure soil NH_4_^+^ and NO_3_^−^ concentrations, we suspended 5 g of soil in a 2 M KCl solution and then measured filtered extracts colorimetrically using a SEAL Automated Segmented Flow analyzer in the Loyola University, Chicago, IL, USA. We measured soil CO_2_ efflux of each sample three times by a closed chamber method in April 2012 as previously described [48], where a LiCOR LI-6400 portable photosynthesis system and LI6400-09 Soil Flux Chamber (LiCOR, Lincoln, NE, USA) were used.

### Statistical analyses

We used a permutation paired *t*-test to determine statistically significant differences between relative abundances of the taxonomic markers, relative abundances of functional genes, and environmental attribute measurements. We compared the composition of microbial communities using three non-parametric dissimilarity tests, including permutational multivariate analysis of variance (Adonis), analysis of similarities (Anosim), and multi-response permutation procedure (Mrpp). Shannon index (H’) was used to estimate the taxonomic or functional gene diversity. We performed detrended correspondence analysis (DCA) to assess overall changes in the microbial community composition and Mantel tests to detect correlations between quantitative measures of microbial community dissimilarity and environmental attributes. To examine the effect of N deposition on individual taxonomic group, functional gene category, or family, we used response ratios to quantify the abundance change by treatment. We used the R package “DESeq2” [49] to calculate the differential abundance (log2-fold change in the relative abundance of each OTU) for N deposition samples as compared with the control samples. We filtered out OTUs that were sparsely represented across all of the samples (i.e., OTUs for which the DESeq2-normalized count across all of the samples (“baseMean”) was < 0.8). Given the high number of mean comparisons and correlations tested, we adjusted the *P*-values with the Benjamini and Hochberg correction method. We considered adjusted *P* < 0.050 to be statistically significant unless otherwise indicated. All statistical analyses were performed by R version 3.0.1 (R Core Team 2013).

## Results

### Taxonomic composition of bacterial communities

The N deposition treatment in the form of Ca(NO_3_)_2_ has been applied twice per year throughout the duration of the experiment. Specifically, 2 g N m^−2^ as a Ca(NO_3_)_2_ solution was applied at the first rains (November) to mimic a periodic N flush, then 5 g m^−2^ N as the slow-release pellet was applied in January to mimic constant atmospheric N deposition [29, 31, 50, 51]. Earlier studies showed that N deposition produced the largest effects on the JRGCE ecosystem [50, 51]. Therefore, we focused on the microbial responses to N deposition applied on top of other climate change drivers after 14 years since the experiment began. The main N effects on microbial communities were largely similar between *n* = 32 and *n* = 4 (see the rest of the “Results” and “Discussion” sections for details), so we used all samples to improve statistical power.

**Fig. 1:**
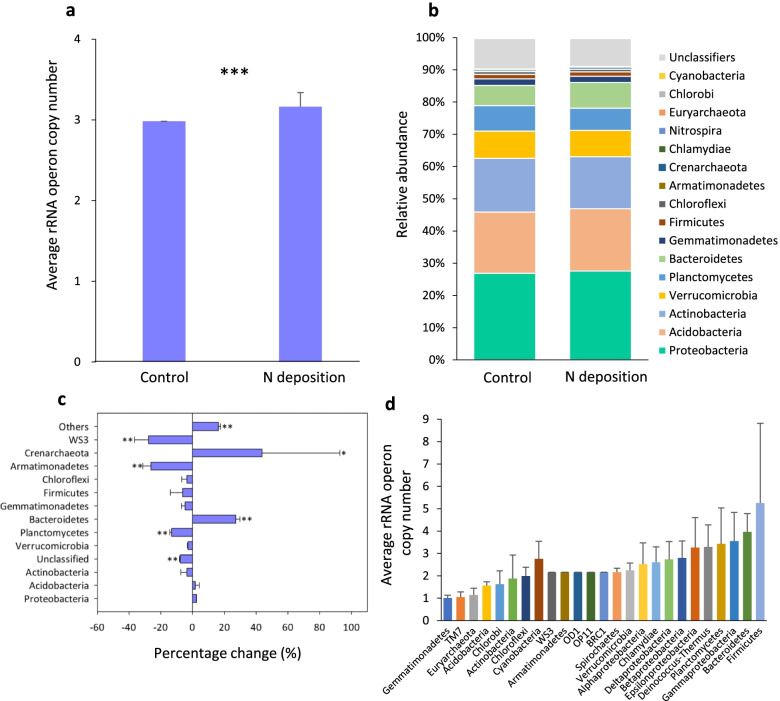
**a** The abundance-weighted average rRNA operon copy number of significantly changed OTUs in control and N deposited samples. **b** Relative abundance of bacterial phylum in control and N deposited samples. **c** The percent change in relative abundances of microbial phyla induced by N deposition. **d** The average estimated rRNA copy number of OTUs derived from each phylum. Error bars indicate standard errors. Asterisks indicate significant differences. *P < 0.050; **P < 0.010

A total of 482,081 sequence reads of the 16S rRNA gene amplicons were obtained from all 64 samples, ranging from 29,275 to 82,066 reads per sample. After resampling at 29,275 sequence reads for all samples, 26,997 operational taxonomic units (OTUs) were generated at the threshold of 97% nucleotide identity. Using three non-parametric statistical analyses (Anosim, Adonis, and Mrpp), we found that N deposition induced significant changes in the overall taxonomic composition of soil bacterial communities (Table 1, Additional file 1: Table S1). Additionally, N deposition increased the estimated abundance-weighted average rRNA operon copy number of significantly changed OTUs for each sample from 2.98 ± 0.21 to 3.16 ± 0.17 (*P* < 0.001, Fig. 1a). However, the α-diversity of the bacterial community was unaltered by N deposition (Additional file 1: Table S2). The responses of the relative abundances of the bacterial phyla to N deposition across the eight factorial combinations of CO_2_, warming, and precipitation (*N* = 32) were generally similar to those observed when considering only N deposition under ambient CO_2_, no warming, and ambient precipitation (*N* = 4, Additional file 1: Fig. S1).

**Table 1:** The effects of N deposition on taxonomic and functional compositions of microbial communities, calculated with Bray-Curtis distance

Statistical approaches		Taxonomic	Functional
Anosim	*R*	0.264	0.048
	*P*^§^	**0.001**^§^	**0.025**
Adonis	*F*	0.074	0.031
	*P*	**0.001**	**0.054**
Mrpp	δ	0.463	0.256
	*P*	**0.001**	**0.035**

^§^Significantly (*P* < 0.050) changed values are shown in bold

The most abundant bacterial taxa were *Proteobacteria*, *Acidobacteria*, *Actinobacteria*, *Verrucomicrobia*, *Planctomycetes*, and *Bacteroidetes*, in decreasing order of relative abundance (Fig. 1b). The relative abundances of *r-Proteobacteria* and *Bacteroidetes* were significantly increased with N deposition (Fig. 1c and Additional file 1: Fig. S2), with a higher estimated rRNA copy number (3.96 ± 0.82 in N deposited samples vs. 3.55 ± 1.29 in control samples, Fig. 1d). In contrast, the relative abundances of *Deltaproteobacteria*, *Planctomycetes*, and most classes of *Acidobacteria* decreased with N deposition (Additional file 1: Fig. S2), with their estimated rRNA copy number (1.56 ± 0.17) being much lower than the average rRNA copy number of all OTUs (2.51 ± 1.28) (Fig. 1d). At the finer taxonomic resolution, relative abundances of *Mycobacterium* and *Anaeromyxobacter* significantly decreased with N deposition, while OTUs affiliated with *Pseudomonas* and 99 out of 124 *Bacteroidetes* OTUs significantly increased (Additional file 1: Table S3).

Regarding nitrifiers, we identified a total of 76 ammonia-oxidizing bacterial OTUs belonging to the genera *Nitrosomonas* (1 OTU) and *Nitrosospira* (75 OTUs) and 77 nitrite-oxidizing bacteria belonging to the genera *Nitrobacter* (48 OTUs) and *Nitrospira* (29 OTUs). Relative abundances of *Nitrosospira* and *Nitrospira* were both significantly increased by N deposition, while the relative abundance of *Nitrobacter* remained unchanged (Additional file 1: Fig. S3).

### Functional composition of microbial community

We detected a total of 60,887 microbial functional genes by GeoChip. Similar to observations at the taxonomic level, N deposition induced significant changes in overall functional compositions of the soil microbial communities (Table 1, Additional file 1: Table S1), but the α-diversity of the soil microbial community based on functional genes remained unaltered (Additional file 1: Table S2). The responses of the relative abundances of the functional genes to N deposition treatment across the eight factorial combinations of CO_2_, warming, and precipitation (*N* = 32) were generally similar to those observed when considering only N deposition under ambient CO_2_, no warming, and ambient precipitation (*N* = 4) (Fig. 1).

#### C cycling

We detected a total of 20,079 genes associated with C cycling. Most of the C fixation genes that significantly responded to N deposition increased in relative abundance except *mcm* encoding methyl malonyl-CoA mutase and *mmce* encoding methyl malonyl-CoA epimerase (associated with 3-hydroxypropionate bicycle), which decreased in relative abundance (Additional file 1: Fig. S4).

**Fig. 2:**
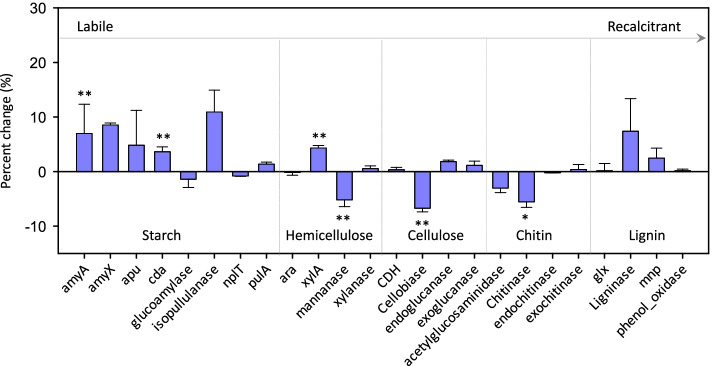
Changes in C cycling gene abundances. The percent change in the relative abundance of C degradation genes by N deposition is calculated as 100*((mean value in N deposited samples/mean value in control samples) – 1). Mean values and standard deviations are presented. Asterisks indicate significant differences. *P < 0.050; **P < 0.010

C degradation genes associated with different substrates showed disparate responses. The relative abundances of *amyA* encoding α-amylase, *cda* encoding cytidine deaminase associated with starch degradation, and *xylA* encoding xylose isomerase associated with hemicellulose degradation increased with N deposition (Fig. 2). In contrast, the relative abundances of *mannanase* gene associated with hemicellulose degradation, *cellobiase* gene associated with cellulose degradation, and *chitinase* gene associated with chitin degradation decreased with N deposition. Genes associated with lignin degradation, such as *glx* encoding glyoxal oxidase, *ligninase*, *mnp* encoding manganese peroxidase, and *phenol oxidase*, remained unchanged. That is, N deposition increased the relative abundances of genes associated with chemically labile C degradation, while it either decreased or had no effect on the relative abundance of genes associated with more chemically recalcitrant C degradation.

The methanogenesis gene *mcrA* decreased with N deposition (Additional file 1: Fig. S5). In contrast, the abundance of the methane-oxidation gene *pmoA* increased with N deposition, while other methane-oxidation genes *mmoX* and *hdrB* remained unchanged.

#### N cycling

**Fig. 3:**
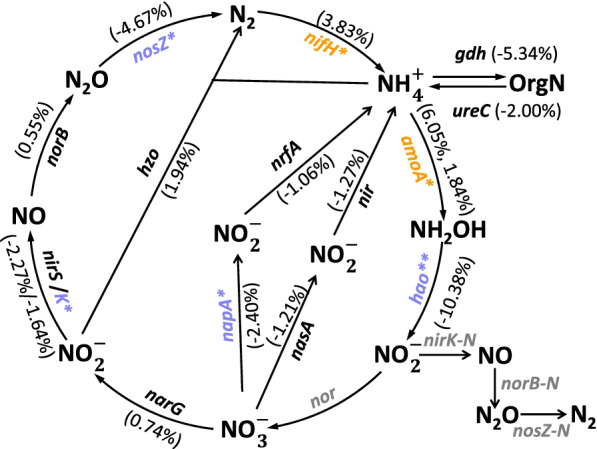
Changes in N cycling gene abundances. The percent change in brackets for each gene is calculated as 100*(( mean value in N deposited samples/mean value in control samples) – 1). Orange and blue represent increases and decreases in gene relative abundance in response to N deposition, respectively. Gray-colored genes are not targeted by GeoChip. Asterisks indicate significant differences. *P < 0.050; **P < 0.010

Relative abundances of several functional genes associated with N cycling were reduced by N deposition (Fig. 3), including *nirK* encoding Cu-like nitrite reductase and denitrification gene *nosZ1* encoding nitrous oxide reductase, along with nitrate reduction gene *napA* encoding nitrate reductase. In contrast, *amoA* encoding ammonia monooxygenase increased in relative abundance with N deposition. The significantly increased *amoA* genes included archaeal *amoA* derived from *Crenarchaeota* (GeneID 218938119, 82570877, 146146994, 164614053, and 124294977) and bacterial *amoA* derived from the *Nitrosomonadaceae* and *Nitrospiraceae* (GeneID 124514869, 161729059, and 78057472) (Additional file 1: Fig. S6). The relative abundance of N_2_ fixation gene *nifH* encoding nitrogenase also increased with N deposition.

#### P cycling

The relative abundance of polyphosphate kinase gene *ppk* associated with polyphosphate synthesis, phytase genes associated with phytic acid hydrolysis, and exopolyphosphatase gene *ppx* associated with polyphosphate degradation remained unchanged (Additional file 1: Fig. S5).

### The network analysis of C and N cycling genes

**Fig. 4:**
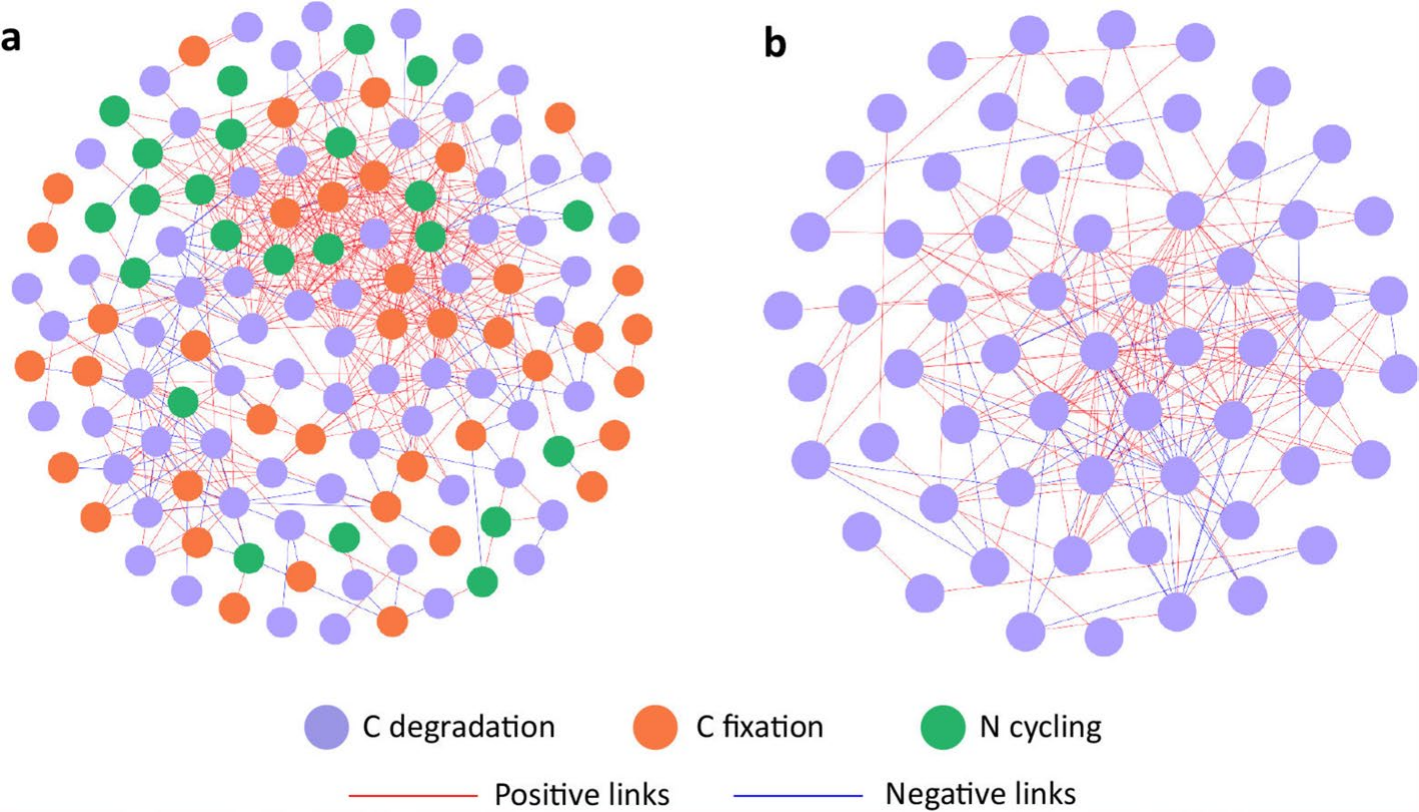
Association networks of microbial functional genes associated with C degradation, C fixation, and N cycling in **a** control samples and **b** N deposited samples. C degradation genes are shown in green circles, C fixation genes are shown in blue circles, and N cycling genes are shown in red circles. Positive links between genes are in red and negative links are in blue

Association networks were constructed to de-convolute complex relationships for functional genes involved in C degradation, C fixation, and N cycling (Fig. 4). The gene networks for both control and N deposited samples had general topological properties of scale-free (power-law *R*^2^ of 0.747~0.914), small world (average path distance of 2.94~3.28), and hierarchy (average clustering coefficient of 0.39~0.51, Additional file 1: Table S4). Although the nodes and links in the network for control samples outnumbered those in the network for N deposited samples (Additional file 1: Table S4), positive links accounted for over half of all links in both networks, suggesting that more microbial C and N cycling genes tended to be co-occurring rather than co-excluding. Although we used the same similarity threshold to construct both networks, topological properties, including average degree and connectedness, were lower in the network of N deposited samples than that of control samples, revealing a less connected network structure. Compared to the network of control samples that contained diverse microbial functional genes associated with C degradation, C fixation, and N cycling, the network of N deposited samples contained only genes associated with C degradation (Fig. 4), suggesting that C degradation genes were intensely connected under N deposition. Consistently, the modularity of the N deposited network was lower than that of the control network (Additional file 1: Table S4), implying fewer coherently changing functional units with N deposition.

### Linkages between environmental attributes and microbial community

Long-term N deposition increased the growth of aboveground biomass and the amount of litter but had no significant effect on belowground biomass (Additional file 1: Table S5). N deposition also increased soil CO_2_ efflux rates in April 2012 from 5.04 to 6.03 μmol m^−2^ s^−1^ (*P* = 0.019). Soil temperature was decreased owing to the shading effect from higher aboveground biomass. Soil pH was increased (*P* = 0.012) from 6.20 to 6.34 by N deposition. Soil NO_3_^−^ was also increased from 79.7 to 612 mg/L and soil NH_4_^+^ was increased from 666 to 1001 mg/L, in addition to the increase of soil TC and TN (*P* = 0.001).

Soil pH and the biomass of annual grasses correlated with quantitative measures of taxonomic composition dissimilarity (Additional file 1: Table S6). By contrast, perennial forb biomass and plant litter correlated with quantitative measures of functional composition dissimilarity. Additionally, soil TC marginally correlated with quantitative measures of both taxonomic and functional composition dissimilarity.

## Discussion

**Fig. 5:**
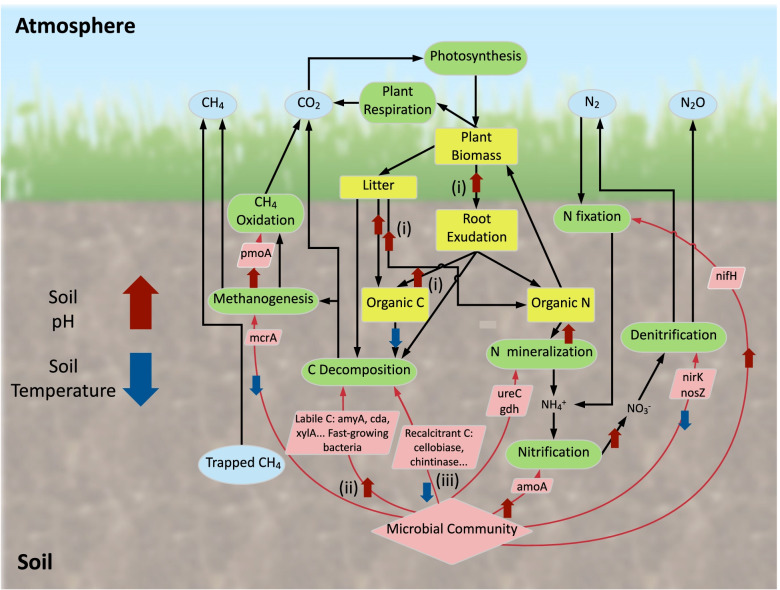
A conceptual model of the effects of N deposition on the terrestrial ecosystem processes. Blue oval frames represent greenhouse gas pools, yellow square frames represent material pools, green frames represent biological processes, pink rhomboid frames represent microbial functional genes, and pink rhombus frames represent microbial communities. Thick black arrows indicate material flows. Microbial mediation of soil biogeochemical process is marked by thin arrows in red and labeled with a “+” or “-” if increases or decreases in gene abundance are observed in this study. “?” represents uncertain microbial feedback. The pink upward arrow indicates the increase of soil pH, and the blue downward arrow indicates the decrease of soil temperature. The (i), (ii), and (iii) mechanisms are labeled near the pathway

In the present study, the observed alteration of soil and plant attributes in response to N deposition led to significant changes in the above- and belowground communities. On the one hand, higher aboveground biomass and litter increased soil nutrient input (mechanism i in Fig. 5). On the other hand, overall taxonomic and functional compositions of the belowground soil bacterial communities were also significantly changed, even when considered across different levels of CO_2_, warming, and precipitation (Table 1 and Additional file 1: Fig. S1). Therefore, the 32 samples used here both improve the statistical power and identify trends that are valid across the range of climate change scenarios tested at Jasper Ridge.

### Long-term N deposition alters the bacterial taxonomic composition

Our results verify previous findings from long-term N amendment experiments in a Minnesota semiarid grassland and Michigan agricultural land showing significant changes in the 16S rRNA gene-based soil microbial community composition [5, 7]. More particularly, the rRNA operon copy number is used as a functional trait related to the maximal bacterial growth rate [52, 53]. Our results concerning the estimated rRNA operon copy number support our hypothesis that N deposition shifts the bacterial community from slow- to fast-growing taxa (Fig. 1) [7]. It is consistent with richer soil nutrients induced by long-term N deposition [54], leading to increased litter mass, soil TC, TN, and NO_3_^−^ concentrations (mechanism ii in Fig. 5 and Additional file 1: Table S5). Similarly, a previous study has shown that ammonium nitrate deposition also decreases the relative abundances of many taxa from *Acidobacteria*, which have low rRNA copy numbers (Fig. 1d) and are thought to be slow-growing [55]. Furthermore, previous reports have shown that ammonium or urea depositions increase the relative abundances of taxa such as *Bacteroidetes* and *r-Proteobacteria* [3, 7, 56] characterized by higher estimated rRNA copy number (Fig. 1d) regarded as fast-growing taxa [57]. Together, it seems that the effects of long-term N deposition on microbial taxonomic composition are independent of N form, likely due to the overall N cycling in the soil-plant system so that the long-term N deposition treatment in an N form may ultimately increase the availability of other N forms as well. For instance, nitrate deposition in the JRGCE has been reported to increase soil ammonium concentrations (this trend was observed here, but the effect was insignificant), reflecting N turnover and increased N mineralization [17, 22, 34].

### The effect of long-term N deposition on microbial functional genes for soil C cycling

N deposition can significantly accelerate chemically labile C decomposition [58, 59]. Consistently, we have observed a significant increase in the relative abundance of the *amyA* gene (Fig. 2), which is among the most abundant genes, providing evidence for the preferential microbial utilization of labile C with N deposition. The significantly increased soil CO_2_ efflux (Additional file 1: Table S5), mediated by higher net primary productivity, was consistent with more labile C [48]. Additionally, N deposition can stabilize or even inhibit the decomposition of chemically recalcitrant C in soil [14, 60]. For instance, N deposition increases the activity of labile C-degrading enzymes but has no effect on or inhibits the activity of lignin-degrading enzymes [11]. Increased use of labile C and decreased use of recalcitrant C can result in soil C accumulation [5, 61]. Here, we observed that N deposition increased the relative abundance of genes associated with chemically labile C degradation but either decreased or had no effect on the relative abundance of genes associated with chemically recalcitrant C degradation (mechanisms ii and iii in Fig. 5). Therefore, N deposition at the JRGCE site is unlikely to induce a priming effect, i.e., fresh C input stimulating recalcitrant old soil organic C degradation [62], which would decrease soil C [63]. Overall, our results reveal a shift in the functional composition of the soil microbial community, which is likely favorable to soil C accumulation.

### The effect of long-term N deposition on microbial functional genes linked to soil N cycling

N deposition has previously been shown to increase potential soil nitrification rate, gross N mineralization, and nitrifier abundances at the JRGCE [16, 17, 30]. Our study shows that some changes in the soil microbial community are consistent with increased nitrification, in particular with higher relative abundances of ammonia-oxidizing bacteria from the genera *Nitrosospira* and *Nitrosomonas* and the nitrite-oxidizing bacteria *Nitrospira* (Additional file 1: Fig. S2). Similarly, N deposition also increases the relative abundances of the *amoA* gene involved in ammonia oxidation (Fig. 3), the rate-limiting step of nitrification in natural grassland [64]. Nitrification is often viewed as a process carried out by phylogenetically restricted microbial groups [65] and as an obligate activity for nitrifiers to derive energy for maintenance and growth. It explains the previous observation that changes in nitrification were associated with the abundances of nitrifiers [66], though such linkage was not detected elsewhere [19]. Generally, the correlation with nitrification holds for nitrifier abundances assessed either by quantitative PCR [67, 68] or by GeoChip [69, 70]. However, it is noticeable that the relative abundances of the *hao* gene encoding hydroxylamine oxidoreductase decreased with N deposition, which could be explained by the fact that conversion of hydroxylamine to nitrite is not the rate-limiting step of nitrification and that a partial decoupling between this step and other steps is possible.

Potential denitrification rates, measured as in situ N_2_O emission from the soil, increase with N deposition at our study site [71]. However, relative abundances of denitrification genes *nirK* and *nosZ* and nitrate reduction gene *napA* decreased with N deposition (Fig. 3), consistent with previous studies unveiling no correlation between denitrification rates and denitrifier abundances [68, 72]. Indeed, denitrification is a facultative process driven mostly by heterotrophs when they are under anaerobic conditions and with sufficient nitrate availability. However, denitrifiers are selected for different reasons than their denitrification capacity under other environmental conditions, often leading to weak activity-abundance relationships [73]. Moreover, the decoupling between denitrification and gene abundance might arise from the saturation of N supply (70 kg ha^−1^ year^−1^ of N) at our study site, since grasslands under Mediterranean climate type are saturated at N loads of 20 kg ha^−1^year^−1^ of N and nitrification and denitrification show nonlinear responses to N supply [74]. Furthermore, denitrification is a broad process carried out by phylogenetically diverse microbial groups [65], which may not be fully represented on GeoChip.

### Long-term N deposition reduces microbial network complexity

Microbial functional genes and the microorganisms that harbor these genes carry out C and N cycling processes [75], and associations among these functional genes could partly, though hardly extensively, be captured by their abundance correlation-based networks [76]. In abundance correlation-based networks of functional genes, correlations can result from multiple mechanisms [77], including (i) metabolic dependence of the linked genes (e.g., links between C and N genes reflecting C/N coupled processes or links between N mineralization and nitrification genes resulting from stepwise N metabolism); (ii) their similar response to environmental variation or substrate availability (e.g., links between different C degradation genes associated with parallel mineralization of soil organic substrates of different molecular forms, or unified response of soil organic matter degradation to edaphic conditions); and/or (iii) their co-dependence on other factors (e.g., soil mineral Fe and Al) instead of direct association. These mechanisms are difficult to be teased apart only based on correlations. In our case, all of the above mechanisms are ecologically meaningful when considering the changes in microbial functional capacities and associations following N deposition. All of these mechanisms are hence potential causes of changes in sample variance that might influence connections and connectedness.

Our results showed that different microbial functional genes associated with C degradation, C fixation, and N cycling formed positive associations in the network of control samples (Fig. 4a), suggesting that microbial C and N processes were likely highly coupled when soil N availability was limited. Considering that growth conditions without N deposition were not optimal, degradation of soil organic matter represented an important source of N to microorganisms. As N availability increases, bacteria become less N and energy (electron acceptor) limited and thus preferentially mineralize progressively more bioavailable soil organic C [78]. Consistently, genes involved in C degradation remained more connected, without clear association with N-related genes (Fig. 4b).

Furthermore, increased N availability could diminish plant dependence on the mycorrhizal supply of organic N monomers [75], leading to more N allocation into plant tissues rather than microbes. As a result, associations among microbial genes involved in N cycling might be reduced. N deposition generally reduces N retention in the soil while increasing N leaching in different ecosystems [79, 80], which could also suppress soil N cycling processes mediated by microbial communities. Our results suggested that soil N availability might have an important role in shaping microbial networks of nutrient cycling and tends to decrease the coupling between N- and C-related functional genes.

### Environmental filters for the soil microbial community

We have identified significant correlations between quantitative measures of microbial community dissimilarity and a set of key soil and plant properties using Mantel tests (Additional file 1: Table S6). The close linkage between soil pH and bacterial community taxonomic composition provides support to a growing body of literature showing that soil pH is the best predictor of soil bacterial diversity and composition, at least from kilometer to continental scales [81–83]. At smaller scales (e.g., in our study), soil pH is rather stable as compared to other soil environmental attributes like moisture or N availability. However, even slight deviations can result in marked changes in microbial communities in response to anthropogenic disturbances that can alter pH (here long-term N deposition) [84, 85].

Our results showed that aboveground plant biomass and litter correlated with quantitative measures of functional composition dissimilarity (Additional file 1: Table S6). Similarly, previous studies in grassland ecosystems have shown that at least 20% of the variation in soil microbial community composition was explained by plant attributes [44, 45]. Much of this variation may be caused by the indirect influence of aboveground plant biomass on microbial communities through enhanced production of dissolved organic C and the promotion of rhizosphere microbial populations [86, 87].

## Conclusions

Overall, our results highlight the importance of investigating the joint responses of microbial functional groups involved in soil C and N dynamics to N availability. In response to long-term N deposition, the soil microbial community shifted toward a higher capacity to use chemically labile C (higher relative abundance of labile C degradation genes) in parallel with a higher relative abundance of fast-growing taxa. In contrast, genes associated with chemically recalcitrant C degradation remained unaltered or decreased. Additionally, the relative abundance of the *amoA* gene, characteristic of the first and rate-limiting step of nitrification in grasslands, increased as did plant biomass, whereas functional genes characterizing the denitrification process decreased with N deposition. It could give rise to a nitrification/denitrification balance favorable for N retention in the plant-soil system. As illustrated in the conceptual model presented in Fig. 5, our results suggest that N deposition favored soil C and N accumulation through three mechanisms: (i) increasing the nutrient source from plants by stimulating plant growth, as shown by increased aboveground biomass and litter; (ii) increasing the potential nutrient availability through the increased relative abundance of nutrient-cycling genes and increased fast-growing bacteria; and (iii) reducing the utilization of chemically recalcitrant C through decreased recalcitrant C degradation genes. Our results demonstrate that a detailed and comprehensive analysis of the response of the soil microbial community, in particular its functional traits and interactions, can help understand and predict the effects of N deposition on soil biogeochemical processes.

## Supplementary Information

**Additional file 1: Table S1.** Comparison of taxonomic and functional β-diversity between and within treatments. **Table S2.** Effects of N deposition on microbial taxonomic and functional diversity, as assessed by Shannon index. **Table S3.** Significantly changed representative OTUs calculated by difference analyses. **Table S4.** Topological properties of microbial functional gene networks. **Table S5.** Summary of soil and vegetation attributes in control and N deposited samples. **Table S6.** Mantel tests for correlations between a range of environmental attributes and quantitative measures of microbial community dissimilarity. **Fig. S1.** Comparison of the percentage change by N deposition for (a) microbial phyla; (b) N cycling genes; and (c) C cycling genes between using 32 and 4 samples as biological replicates. **Fig. S2.** The percentage change in relative abundances of microbial class induced by long-term N deposition. Asterisks indicate significant differences. *, *P* < 0.050; **, *P* < 0.010. **Fig. S3.** The percentage change in the relative abundance of major microbial genera induced by long-term N deposition treatment. All selected genera are significantly changed by N deposition treatment as calculated by the response ratio analysis. **Fig. S4.** The percentage change in the relative abundance of genes associated with C fixation induced by N deposition, calculated as 100*(( mean value in N deposited samples/mean value in control samples) – 1). Mean values and standard deviations are presented. Asterisks indicate significant differences. *, *P* < 0.050; **, *P* < 0.010. The numbers in the figure represent the pathways of C fixation. (i) 3-hydroxypropionate bicycle, (ii) Bacterial microcompartments, (iii) Calvin cycle, and (iv) Reductive tricarboxylic acid cycle. **Fig. S5.** The percentage change in the relative abundance of genes associated with methane and phosphorus cycling genes induced by N deposition, calculated as 100*((mean value in N deposited samples/mean value in control samples) – 1). Mean values and standard deviations are presented. Asterisks indicate significant differences. *, *P* < 0.050; **, *P* < 0.010. **Fig. S6.** N deposition effects on *amoA* gene. The relative abundance of *amoA* is presented as the signal intensity difference between control and N deposited samples. Error bars represent standard errors. Blue bars represent genes derived from archaea (AOA), and pink bars represent genes derived from bacteria (AOB). Asterisks indicate significant differences. *, *P* < 0.050; **, *P* < 0.010.

## Data Availability

GeoChip data are available online (www.ncbi.nlm.nih.gov/geo/) with the accession number GSE107168. MiSeq data are available in the NCBI SRA database with the accession number SRP126539.

## Abbreviations

N: Nitrogen
C: Carbon
rRNA: Ribosomal ribonucleic acid
JRGCE: Jasper Ridge Global Change Experiment
DNA: Deoxyribonucleic acid
PCR: Polymerase chain reaction
SNR: Signal-to-noise ratio
RMT: Random matrix theory
St: Similarity threshold
avgK: Average degree
CD: Centralization of degree
avgCC: Average clustering coefficient
HD: Harmonic geodesic distance
CB: Centralization of betweenness
CS: Centralization of stress centrality
AG: Annual grasses
PG: Perennial grasses
AF: Annual forbs
AP: Perennial forbs
TC: Total soil carbon
TN: Total soil nitrogen
Anosim: Analysis of similarities
Mrpp: Multi-response permutation procedure
DCA: Detrended correspondence analysis
OTUs: Operational taxonomic units
CO_2_: Carbon dioxide
N_2_O: Nitrous oxide
