# Correction: Long-term nitrogen deposition enhances microbial capacities in soil carbon stabilization but reduces network complexity

**DOI:** 10.1186/s40168-022-01349-1

**Published:** 2022-09-03

**Authors:** Xingyu Ma, Tengxu Wang, Zhou Shi, Nona R. Chiariello, Kathryn Docherty, Christopher B. Field, Jessica Gutknecht, Qun Gao, Yunfu Gu, Xue Guo, Bruce A. Hungate, Jiesi Lei, Audrey Niboyet, Xavier Le Roux, Mengting Yuan, Tong Yuan, Jizhong Zhou, Yunfeng Yang

**Affiliations:** 1 State Key Joint Laboratory of Environment Simulation and Pollution Control, School of Environment, Tsinghua University, Beijing, 100084 China; 2 China Urban Construction Design & Research Institute Co., Ltd, Beijing, 100120 China; 3 North China Municipal Engineering Design & Research Institute Co., Ltd., the Beijing Branch, Beijing, 100081 China; 4 Institute for Environmental Genomics and Department of Microbiology and Plant Biology, University of Oklahoma, Norman, OK 73019 USA; 5 Department of Global Ecology, Carnegie Institution for Science, Stanford, CA 94305 USA; 6 Department of Biological Sciences, Western Michigan University, Kalamazoo, MI 49008 USA; 7 Department of Soil Ecology, Helmholtz Centre for Environmental Research – UFZ, 06120 Halle, Germany; 8 Present Address: Department of Soil, Water, and Climate, University of Minnesota, Twin Cities, Saint Paul, MN 55104 USA; 9 Department of Microbiology, College of Resource, Sichuan Agricultural University, Chengdu, 611130 China; 10 Center for Ecosystem Science and Society, and Department of Biological Sciences, Northern Arizona University, Flagstaf, AZ 86011 USA; 11 Sorbonne Université, Université Paris Cité, UPEC, CNRS, INRAE, IRD, Institut d’Ecologie et des Sciences de l’Environnement de Paris, iEES-Paris, Paris, France; 12 AgroParisTech, Paris, France; 13 Microbial Ecology Centre LEM, INRAE, CNRS, University of Lyon, University Lyon 1, VetAgroSup, UMR INRAE 1418, 43 boulevard du 11 novembre 1918, 69622 Villeurbanne, France; 14 Department of Environmental Science, Policy and Management, University of California Berkeley, Berkeley, CA 94720 USA; 15 Earth Sciences Division, Lawrence Berkeley National Laboratory, Berkeley, CA 94720 USA

**Correction: Microbiome 10, 112 (2022)**

**https://doi.org/10.1186/s40168-022-01309-9**

Following the publication of the original article [1], the author reported that the request to remove affiliation 1 from the author “Jizhong Zhou” was not implemented.

This has been corrected in this correction article and the original article has been updated.

## References

[1] Ma X, Wang T, Shi Z (2022). Long-term nitrogen deposition enhances microbial capacities in soil carbon stabilization but reduces network complexity. Microbiome.

