# Supplementary material for: Long-term nitrogen deposition enhances microbial capacities in soil carbon stabilization but reduces network complexity
Source: Microbiome. 2022 Jul 28;10:112. doi: 10.1186/s40168-022-01309-9 (PMC9330674; doi:10.1186/s40168-022-01309-9)

**Supplemental Materials**

**Table S1.** Comparison of taxonomic and functional β-diversity between and within treatments

|  | Distance algorithm | Treatment | Distance | Std. | ANOVA |
| --- | --- | --- | --- | --- | --- |
| Taxonomic β-diversity | Bray | aN - aN^*^ | 0.470 | 0.053 | b^#^ |
|  |  | eN - aN | 0.490 | 0.060 | a |
|  |  | eN - eN | 0.456 | 0.040 | c |
|  | Sorensen  (unweighted bray) | aN - aN | 0.540 | 0.040 | b |
|  |  | eN - aN | 0.546 | 0.045 | a |
|  |  | eN - eN | 0.524 | 0.033 | c |
|  | Ruzicka  (weighted Jaccard) | aN - aN | 0.638 | 0.048 | b |
|  |  | eN - aN | 0.655 | 0.053 | a |
|  |  | eN - eN | 0.625 | 0.038 | c |
|  | Jaccard  (unweighted Jaccard) | aN - aN | 0.701 | 0.034 | a |
|  |  | eN - aN | 0.705 | 0.037 | a |
|  |  | eN - eN | 0.687 | 0.028 | b |
| Functional β-diversity | Bray | aN - aN | 0.242 | 0.084 | b |
|  |  | eN - aN | 0.262 | 0.083 | b |
|  |  | eN - eN | 0.270 | 0.086 | a |
|  | Sorensen  (unweighted bray) | aN - aN | 0.197 | 0.055 | b |
|  |  | eN - aN | 0.210 | 0.054 | b |
|  |  | eN - eN | 0.215 | 0.056 | a |
|  | Ruzicka  (weighted Jaccard) | aN - aN | 0.383 | 0.103 | b |
|  |  | eN - aN | 0.408 | 0.098 | b |
|  |  | eN - eN | 0.418 | 0.101 | a |
|  | Jaccard  (unweighted Jaccard) | aN - aN | 0.326 | 0.074 | b |
|  |  | eN - aN | 0.345 | 0.071 | b |
|  |  | eN - eN | 0.350 | 0.073 | a |

^*^aN: control samples; eN: N deposited samples.

^#^*P*-values were adjusted by Bonferroni correction, in which *P*-values were multiplied by the number of comparisons. Different alphabets mean significant differences.

**Table S2.** Effects of N deposition on microbial taxonomic and functional diversity, as assessed by Shannon index.

|  | Control samples | N deposited samples | *P*-value |
| --- | --- | --- | --- |
| Taxonomic diversity | 8.21 | 8.19 | 0.480 |
| Functional diversity | 10.42 | 10.36 | 0.200 |

**Table S3.** Significantly changed representative OTUs calculated by difference analyses.

| OTU | Phylum | Genus | BaseMean | log_2_FoldChange | Adjust *P*^§^ |
| --- | --- | --- | --- | --- | --- |
| Increased OTU |  |  |  |  |  |
| OTU_59267 | *Proteobacteria* | *Pseudomonas* | 2.14 | 1.86 | 0.001 |
| OTU_541 | *Proteobacteria* | *Pseudomonas* | 0.81 | 1.77 | 0.016 |
| OTU_29947 | *Bacteroidetes* | *Ohtaekwangia* | 6.58 | 1.71 | 0.000 |
| OTU_23187 | *Bacteroidetes* | *Ohtaekwangia* | 3.74 | 1.70 | 0.049 |
| OTU_34480 | *Bacteroidetes* | *Ohtaekwangia* | 2.15 | 1.54 | 0.019 |
| OTU_17675 | *Bacteroidetes* | *Ohtaekwangia* | 1.93 | 1.34 | 0.027 |
| OTU_18113 | *Bacteroidetes* | *Ohtaekwangia* | 2.84 | 1.34 | 0.009 |
| OTU_85448 | *Bacteroidetes* | *Ohtaekwangia* | 5.06 | 1.30 | 0.001 |
| OTU_79487 | *Bacteroidetes* | *Ohtaekwangia* | 1.23 | 1.28 | 0.041 |
| OTU_25095 | *Bacteroidetes* | *Ohtaekwangia* | 1.08 | 1.27 | 0.038 |
| OTU_18853 | *Bacteroidetes* | *Ohtaekwangia* | 3.13 | 0.78 | 0.047 |
| OTU_41997 | *Bacteroidetes* | *Ohtaekwangia* | 11.32 | 0.74 | 0.019 |
| OTU_13142 | *Bacteroidetes* | *Ohtaekwangia* | 22.77 | 0.65 | 0.009 |
| OTU_19070 | *Bacteroidetes* | Unclassified | 1.67 | 1.03 | 0.034 |
| OTU_26226 | *Bacteroidetes* | *Fluviicola* | 0.84 | 1.28 | 0.049 |
| OTU_18080 | *Bacteroidetes* | Unclassified | 0.85 | 1.90 | 0.003 |
| OTU_106695 | *Bacteroidetes* | Unclassified | 1.70 | 1.80 | 0.003 |
| OTU_29992 | *Bacteroidetes* | *Flavobacterium* | 1.82 | 3.40 | 0.000 |
| OTU_11281 | *Bacteroidetes* | *Flavobacterium* | 1.39 | 2.98 | 0.000 |
| OTU_19061 | *Bacteroidetes* | *Flavobacterium* | 0.89 | 2.66 | 0.002 |
| OTU_48464 | *Bacteroidetes* | *Flavobacterium* | 0.93 | 1.94 | 0.019 |
| OTU_28836 | *Bacteroidetes* | *Flavobacterium* | 2.81 | 1.77 | 0.002 |
| OTU_84843 | *Bacteroidetes* | *Flavobacterium* | 3.03 | 1.70 | 0.016 |
| OTU_76917 | *Bacteroidetes* | *Flavobacterium* | 6.17 | 1.60 | 0.000 |
| OTU_126479 | *Bacteroidetes* | *Flavobacterium* | 0.92 | 1.54 | 0.020 |
| OTU_89477 | *Bacteroidetes* | *Flavobacterium* | 2.60 | 1.28 | 0.014 |
| OTU_19969 | *Bacteroidetes* | *Flavobacterium* | 1.99 | 1.13 | 0.025 |
| OTU_26206 | *Bacteroidetes* | *Flavobacterium* | 27.53 | 1.01 | 0.004 |
| OTU_129324 | *Bacteroidetes* | *Flavobacterium* | 5.95 | 0.99 | 0.004 |
| OTU_17095 | *Bacteroidetes* | *Flavobacterium* | 12.03 | 0.91 | 0.015 |
| OTU_16850 | *Bacteroidetes* | *Flavobacterium* | 18.79 | 0.59 | 0.016 |
| OTU_30355 | *Bacteroidetes* | Unclassified | 1.96 | 1.92 | 0.001 |
| OTU_88250 | *Bacteroidetes* | *Chitinophaga* | 0.87 | 2.37 | 0.000 |
| OTU_31009 | *Bacteroidetes* | *Ferruginibacter* | 3.53 | 3.00 | 0.000 |
| OTU_112311 | *Bacteroidetes* | *Ferruginibacter* | 4.95 | 1.50 | 0.000 |
| OTU_18079 | *Bacteroidetes* | *Ferruginibacter* | 2.41 | 1.46 | 0.009 |
| OTU_113080 | *Bacteroidetes* | *Ferruginibacter* | 2.30 | 1.44 | 0.001 |
| OTU_44662 | *Bacteroidetes* | *Ferruginibacter* | 6.16 | 1.30 | 0.000 |
| OTU_39127 | *Bacteroidetes* | *Ferruginibacter* | 1.28 | 1.23 | 0.016 |
| OTU_55603 | *Bacteroidetes* | *Ferruginibacter* | 2.65 | 0.90 | 0.027 |
| OTU_25973 | *Bacteroidetes* | *Ferruginibacter* | 4.39 | 0.80 | 0.039 |
| OTU_48613 | *Bacteroidetes* | *Ferruginibacter* | 17.27 | 0.78 | 0.002 |
| OTU_16990 | *Bacteroidetes* | *Flavitalea* | 7.21 | 1.14 | 0.018 |
| OTU_18074 | *Bacteroidetes* | *Niabella* | 3.05 | 1.33 | 0.023 |
| OTU_19334 | *Bacteroidetes* | *Niastella* | 12.33 | 1.27 | 0.000 |
| OTU_56646 | *Bacteroidetes* | *Sediminibacterium* | 6.48 | 0.71 | 0.007 |
| OTU_117823 | *Bacteroidetes* | *Segetibacter* | 5.12 | 0.86 | 0.009 |
| OTU_9280 | *Bacteroidetes* | *Terrimonas* | 4.27 | 2.52 | 0.000 |
| OTU_25305 | *Bacteroidetes* | *Terrimonas* | 2.57 | 1.53 | 0.000 |
| OTU_161598 | *Bacteroidetes* | *Terrimonas* | 1.71 | 1.50 | 0.020 |
| OTU_33709 | *Bacteroidetes* | *Terrimonas* | 3.52 | 0.96 | 0.001 |
| OTU_21521 | *Bacteroidetes* | *Terrimonas* | 12.61 | 0.88 | 0.045 |
| OTU_24513 | *Bacteroidetes* | Unclassified | 1.63 | 1.97 | 0.000 |
| OTU_34668 | *Bacteroidetes* | Unclassified | 4.23 | 1.95 | 0.000 |
| OTU_18296 | *Bacteroidetes* | Unclassified | 1.10 | 1.83 | 0.005 |
| OTU_85176 | *Bacteroidetes* | Unclassified | 1.06 | 1.81 | 0.012 |
| OTU_16931 | *Bacteroidetes* | Unclassified | 8.60 | 1.62 | 0.000 |
| OTU_22589 | *Bacteroidetes* | Unclassified | 6.03 | 1.58 | 0.002 |
| OTU_18742 | *Bacteroidetes* | Unclassified | 2.15 | 1.48 | 0.008 |
| OTU_89651 | *Bacteroidetes* | Unclassified | 9.82 | 1.35 | 0.001 |
| OTU_143142 | *Bacteroidetes* | Unclassified | 6.69 | 1.28 | 0.003 |
| OTU_73044 | *Bacteroidetes* | Unclassified | 3.38 | 1.21 | 0.001 |
| OTU_17204 | *Bacteroidetes* | Unclassified | 1.16 | 1.21 | 0.048 |
| OTU_18127 | *Bacteroidetes* | Unclassified | 2.01 | 1.19 | 0.021 |
| OTU_19091 | *Bacteroidetes* | Unclassified | 9.62 | 1.02 | 0.000 |
| OTU_19982 | *Bacteroidetes* | Unclassified | 2.51 | 0.92 | 0.028 |
| OTU_30812 | *Bacteroidetes* | Unclassified | 7.91 | 0.89 | 0.006 |
| OTU_72139 | *Bacteroidetes* | Unclassified | 8.73 | 0.79 | 0.005 |
| OTU_76350 | *Bacteroidetes* | Unclassified | 82.49 | 0.57 | 0.003 |
| OTU_105375 | *Bacteroidetes* | Unclassified | 24.08 | 0.50 | 0.003 |
| OTU_17090 | *Bacteroidetes* | *Adhaeribacter* | 6.94 | 1.36 | 0.001 |
| OTU_41270 | *Bacteroidetes* | *Adhaeribacter* | 18.89 | 0.74 | 0.001 |
| OTU_25503 | *Bacteroidetes* | *Adhaeribacter* | 10.99 | 0.67 | 0.045 |
| OTU_18230 | *Bacteroidetes* | *Cytophaga* | 11.27 | 1.39 | 0.001 |
| OTU_4372 | *Bacteroidetes* | *Sporocytophaga* | 0.80 | 1.37 | 0.039 |
| OTU_33961 | *Bacteroidetes* | *Cesiribacter* | 1.34 | 1.38 | 0.024 |
| OTU_76402 | *Bacteroidetes* | *Fabibacter* | 1.31 | 1.50 | 0.004 |
| OTU_18259 | *Bacteroidetes* | Unclassified | 2.99 | 1.07 | 0.023 |
| OTU_18321 | *Bacteroidetes* | *Pedobacter* | 1.06 | 1.93 | 0.001 |
| OTU_26812 | *Bacteroidetes* | Unclassified | 1.11 | 2.32 | 0.001 |
| OTU_21016 | *Bacteroidetes* | Unclassified | 5.24 | 3.03 | 0.000 |
| OTU_29297 | *Bacteroidetes* | Unclassified | 1.32 | 2.38 | 0.001 |
| OTU_40756 | *Bacteroidetes* | Unclassified | 1.15 | 1.16 | 0.025 |
| OTU_21809 | *Bacteroidetes* | Unclassified | 1.57 | 1.08 | 0.023 |
| OTU_62337 | *Bacteroidetes* | Unclassified | 11.29 | 0.95 | 0.038 |
| OTU_18471 | *Bacteroidetes* | Unclassified | 16.94 | 0.58 | 0.005 |
| OTU_119048 | *Bacteroidetes* | Unclassified | 1.38 | 3.08 | 0.000 |
| OTU_97392 | *Bacteroidetes* | Unclassified | 2.68 | 2.11 | 0.000 |
| OTU_19521 | *Bacteroidetes* | Unclassified | 1.13 | 1.78 | 0.003 |
| OTU_10064 | *Bacteroidetes* | Unclassified | 29.54 | 1.73 | 0.000 |
| OTU_19963 | *Bacteroidetes* | Unclassified | 1.62 | 1.66 | 0.003 |
| OTU_72001 | *Bacteroidetes* | Unclassified | 1.39 | 1.59 | 0.001 |
| OTU_100887 | *Bacteroidetes* | Unclassified | 1.60 | 1.53 | 0.004 |
| OTU_31229 | *Bacteroidetes* | Unclassified | 3.76 | 1.46 | 0.000 |
| OTU_46231 | *Bacteroidetes* | Unclassified | 2.33 | 1.22 | 0.001 |
| OTU_20516 | *Bacteroidetes* | Unclassified | 7.99 | 1.17 | 0.000 |
| OTU_33281 | *Bacteroidetes* | Unclassified | 10.66 | 1.02 | 0.000 |
| OTU_52833 | *Bacteroidetes* | Unclassified | 1.89 | 1.01 | 0.036 |
| OTU_21922 | *Bacteroidetes* | Unclassified | 2.21 | 1.01 | 0.029 |
| OTU_21989 | *Bacteroidetes* | Unclassified | 2.93 | 0.76 | 0.040 |
| OTU_74737 | *Bacteroidetes* | Unclassified | 16.57 | 0.58 | 0.048 |
| Decreased OTU |  |  |  |  |  |
| OTU_52819 | *Bacteroidetes* | *Ohtaekwangia* | 1.50 | -1.48 | 0.032 |
| OTU_42440 | *Bacteroidetes* | *Ohtaekwangia* | 7.80 | -0.51 | 0.049 |
| OTU_15251 | *Bacteroidetes* | Unclassified | 5.57 | -1.68 | 0.001 |
| OTU_17111 | *Bacteroidetes* | Unclassified | 4.15 | -0.92 | 0.012 |
| OTU_18664 | *Bacteroidetes* | *Flavisolibacter* | 3.76 | -1.22 | 0.000 |
| OTU_121490 | *Bacteroidetes* | *Flavitalea* | 5.84 | -1.28 | 0.001 |
| OTU_19791 | *Bacteroidetes* | *Hydrotalea* | 1.59 | -1.09 | 0.023 |
| OTU_17536 | *Bacteroidetes* | *Niastella* | 1.75 | -1.10 | 0.044 |
| OTU_105475 | *Bacteroidetes* | *Niastella* | 2.86 | -0.90 | 0.015 |
| OTU_136639 | *Bacteroidetes* | *Sediminibacterium* | 1.45 | -2.12 | 0.001 |
| OTU_37300 | *Bacteroidetes* | *Sediminibacterium* | 2.49 | -0.87 | 0.040 |
| OTU_30027 | *Bacteroidetes* | Unclassified | 1.75 | -1.44 | 0.004 |
| OTU_23626 | *Bacteroidetes* | Unclassified | 0.90 | -1.36 | 0.029 |
| OTU_42664 | *Bacteroidetes* | Unclassified | 1.39 | -1.27 | 0.016 |
| OTU_294 | *Bacteroidetes* | Unclassified | 2.24 | -1.04 | 0.020 |
| OTU_18642 | *Bacteroidetes* | Unclassified | 5.36 | -0.90 | 0.007 |
| OTU_22058 | *Bacteroidetes* | Unclassified | 3.29 | -0.79 | 0.024 |
| OTU_18654 | *Bacteroidetes* | Unclassified | 22.97 | -0.68 | 0.001 |
| OTU_27943 | *Bacteroidetes* | *Cytophaga* | 0.94 | -3.38 | 0.000 |
| OTU_19490 | *Bacteroidetes* | *Cytophaga* | 1.10 | -1.27 | 0.031 |
| OTU_4196 | *Bacteroidetes* | *Sporocytophaga* | 2.39 | -1.36 | 0.001 |
| OTU_43195 | *Bacteroidetes* | *Mucilaginibacter* | 1.05 | -1.48 | 0.020 |
| OTU_120 | *Bacteroidetes* | *Mucilaginibacter* | 1.16 | -1.45 | 0.010 |
| OTU_18163 | *Bacteroidetes* | Unclassified | 4.95 | -2.30 | 0.000 |
| OTU_21842 | *Bacteroidetes* | Unclassified | 3.88 | -0.84 | 0.014 |
| OTU_43427 | *Actinobacteria* | *Mycobacterium* | 0.92 | -1.27 | 0.041 |
| OTU_25203 | *Actinobacteria* | *Mycobacterium* | 1.11 | -1.24 | 0.022 |
| OTU_17016 | *Proteobacteria* | *Anaeromyxobacter* | 30.54 | -0.98 | 0.002 |
| OTU_111042 | *Proteobacteria* | *Anaeromyxobacter* | 2.15 | -0.93 | 0.045 |
| OTU_3076 | *Proteobacteria* | *Anaeromyxobacter* | 81.21 | -0.92 | 0.001 |
| OTU_43651 | *Proteobacteria* | *Anaeromyxobacter* | 17.69 | -0.41 | 0.047 |

^§^*P*-values were adjusted with the Benjamini and Hochberg correction method.**Table S4.** Topological properties of microbial functional gene networks

| Network properties | Control samples | N deposited samples |
| --- | --- | --- |
| Total nodes | 130 | 61 |
| Total links | 548 | 207 |
| Positive links (%) | 83 | 77 |
| Negative links (%) | 17 | 23 |
| R square of power-law | 0.91 | 0.75 |
| Modularity | 0.40 | 0.33 |
| Average degree (avgK) | 8.43 | 6.79 |
| Average clustering coefficient (avgCC) | 0.39 | 0.51 |
| Average path distance (GD) | 3.28 | 2.94 |
| Geodesic efficiency (E) | 0.37 | 0.43 |
| Harmonic geodesic distance (HD) | 2.68 | 2.31 |
| Maximal degree | 37 | 26 |
| Nodes with max degree | acetyl_CoA_carboxylase | cutinase |
| Centralization of degree (CD) | 0.23 | 0.33 |
| Maximal betweenness | 1178.31 | 337.05 |
| Nodes with max betweenness | nosZ | cutinase |
| Centralization of betweenness (CB) | 0.13 | 0.16 |
| Maximal stress centrality | 8182 | 1407 |
| Nodes with max stress centrality | nosZ | cutinase |
| Centralization of stress centrality (CS) | 0.88 | 0.67 |
| Maximal eigenvector centrality | 0.28 | 0.33 |
| Nodes with max eigenvector centrality | acetyl_CoA_carboxylase | cutinase |
| Centralization of eigenvector centrality (CE) | 0.23 | 0.25 |
| Density (D) | 0.07 | 0.11 |
| Transitivity (Trans) | 0.47 | 0.51 |
| Connectedness (Con) | 0.94 | 0.91 |
| Efficiency | 0.94 | 0.89 |

**Table S5.** Summary of soil and vegetation attributes in control and N deposited samples

| Environmental attributes | Control samples | N deposited samples | *P*-value |
| --- | --- | --- | --- |
| Soil attributes |  |  |  |
| Temperature (^o^C) | 16.13 (0.97)^*^ | 15.55 (0.96) | **0.001**^†^ |
| pH | 6.20 (0.18) | 6.34 (0.23) | **0.005** |
| Moisture Fraction (%) | 8.52 (2.01) | 8.35 (2.08) | 0.732 |
| NO_3_^-^ (mg/L) | 79.7 (44.24) | 612 (337.67) | **0.001** |
| NH_4_^+^ (mg/L) | 666 (128) | 1001 (461) | 0.349 |
| TC (%)^§^ | 1.23 (0.19) | 1.39 (0.22) | **0.001** |
| TN (%) | 0.12 (0.01) | 0.13 (0.02) | **0.001** |
| Soil CO_2_ efflux (μmol m^-2^ s^-1^) | 5.04 (1.67) | 6.03 (2.13) | **0.019** |
| Vegetation attributes  (g/141cm^-2^) |  |  |  |
| AG | 2.22 (0.96) | 2.90 (1.71) | **0.050** |
| AF | 1.26 (0.78) | 3.97 (3.83) | **0.001** |
| PF | 0.54 (0.51) | 0.74 (0.41) | 0.429 |
| PG | 0.32 (0.02) | 0.38 (0.02) | 0.853 |
| Total aboveground biomass | 4.35 (1.34) | 7.99 (3.27) | **0.001** |
| Litter | 2.00 (1.24) | 2.69 (1.96) | 0.117 |
| Belowground biomass | 0.21 (0.15) | 0.16 (0.09) | 0.118 |

^§^Abbreviations**:** TC—total C; TN—total N; AG—annual grass biomass; AF—annual forb biomass; PF—perennial forb biomass; PG —perennial grass biomass.

^†^*P*-value means the main effect of N deposition calculated by ANOVA. Factors including experimental block, elevated CO_2_, warming, nitrate deposition, and enhanced precipitation were all considered in the ANOVA model. Significant (*P* < 0.050) values are shown in bold.

^*^The value in the bracket is the standard deviation.

**Table S6.** Mantel tests for correlations between a range of environmental attributes and quantitative measures of microbial community dissimilarity

|  | Taxonomic | | Functional | |
| --- | --- | --- | --- | --- |
|  | *r* | *P* | *r* | *P* |
| Soil attributes |  |  |  |  |
| Temperature (^o^C) | 0.002 | 0.46 | -0.035 | 0.74 |
| pH | 0.245 | **0.001**^†^ | -0.128 | 0.99 |
| Moisture Fraction (%) | 0.052 | 0.20 | -0.075 | 0.88 |
| NO_3_^-^ (mg/L) | 0.081 | 0.16 | 0.037 | 0.28 |
| NH_4_^+^ (mg/L) | -0.120 | 0.95 | -0.033 | 0.55 |
| TC (%)^§^ | 0.082 | 0.07 | 0.091 | 0.09 |
| TN (%) | 0.013 | 0.40 | 0.136 | 0.07 |
| Vegetation attributes |  |  |  |  |
| AG | 0.183 | **0.01** | -0.007 | 0.49 |
| AF | -0.023 | 0.57 | 0.019 | 0.36 |
| PF | -0.023 | 0.59 | 0.302 | **0.02** |
| PG | -0.064 | 0.74 | -0.054 | 0.68 |
| Aboveground biomass | 0.023 | 0.34 | 0.125 | 0.08 |
| Litter | 0.023 | 0.34 | 0.313 | **0.01** |
| Belowground biomass | 0.125 | 0.11 | -0.082 | 0.83 |

^§^Abbreviations: TC—total C; TN—total N; AG—annual grass biomass; AF—annual forb biomass; PF—perennial forb biomass; PG—perennial grass biomass.

^†^Significant (*P* < 0.050) correlations are shown in bold.

**Fig. S1** Comparison of the percentage change by N deposition for (a) microbial phyla; (b) N cycling genes; and (c) C cycling genes between using 32 and 4 samples as biological replicates.

**Fig. S2** The percentage change in relative abundances of microbial class induced by long-term N deposition. Asterisks indicate significant differences. *, *P* < 0.050; **, *P* < 0.010.


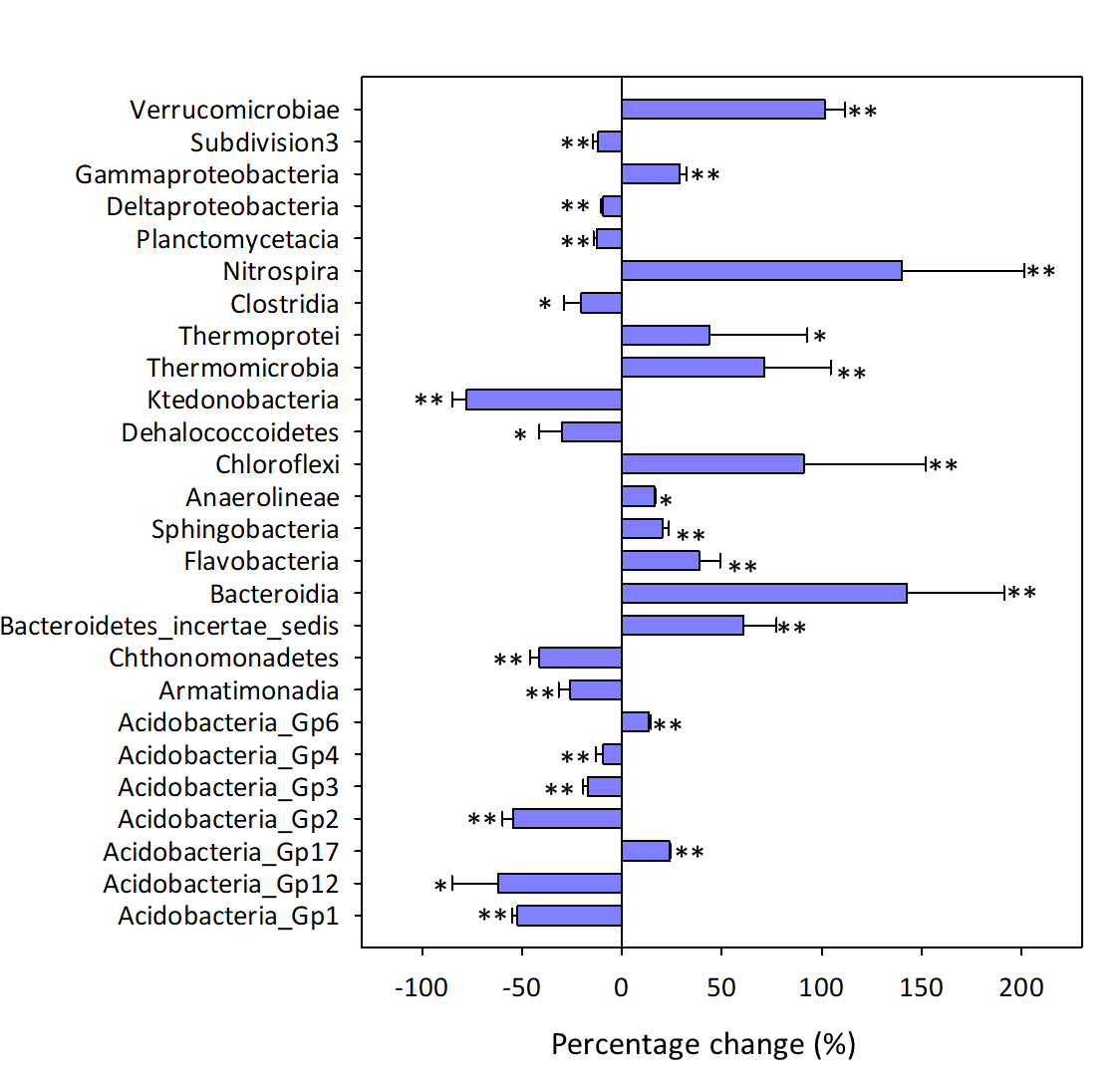


**Fig. S3** The percentage change in the relative abundance of major microbial genera induced by long-term N deposition treatment. All selected genera are significantly changed by N deposition treatment as calculated by the response ratio analysis.


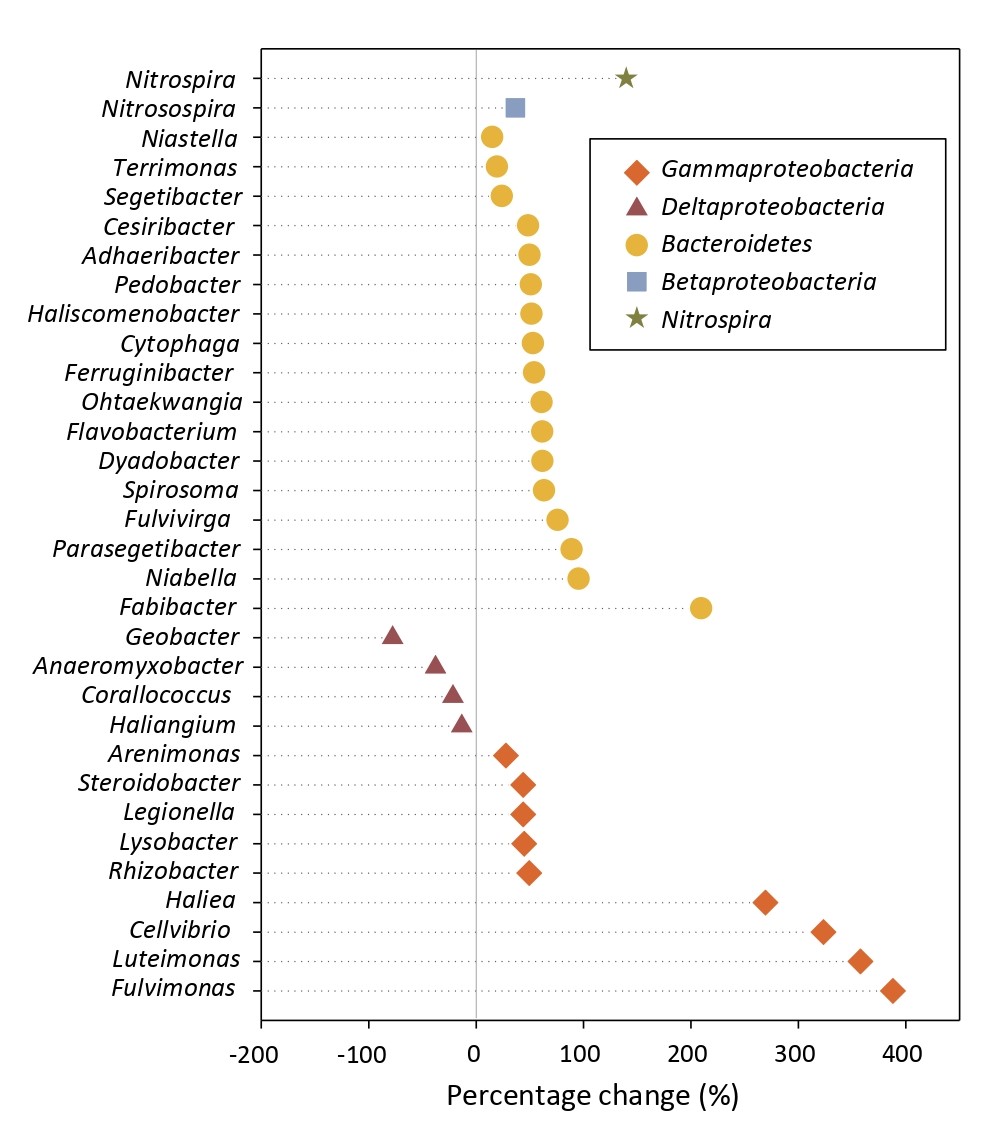


**Fig. S4** The percentage change in the relative abundance of genes associated with C fixation induced by N deposition, calculated as 100*(( mean value in N deposited samples/mean value in control samples) ‒ 1). Mean values and standard deviations are presented. Asterisks indicate significant differences. *, *P* < 0.050; **, *P* < 0.010. The numbers in the figure represent the pathways of C fixation. (i) 3-hydroxypropionate bicycle, (ii) Bacterial microcompartments, (iii) Calvin cycle, and (iv) Reductive tricarboxylic acid cycle.

**Fig. S5** The percentage change in the relative abundance of genes associated with methane and phosphorus cycling genes induced by N deposition, calculated as 100*((mean value in N deposited samples/mean value in control samples) ‒ 1). Mean values and standard deviations are presented. Asterisks indicate significant differences. *, *P* < 0.050; **, *P* < 0.010.


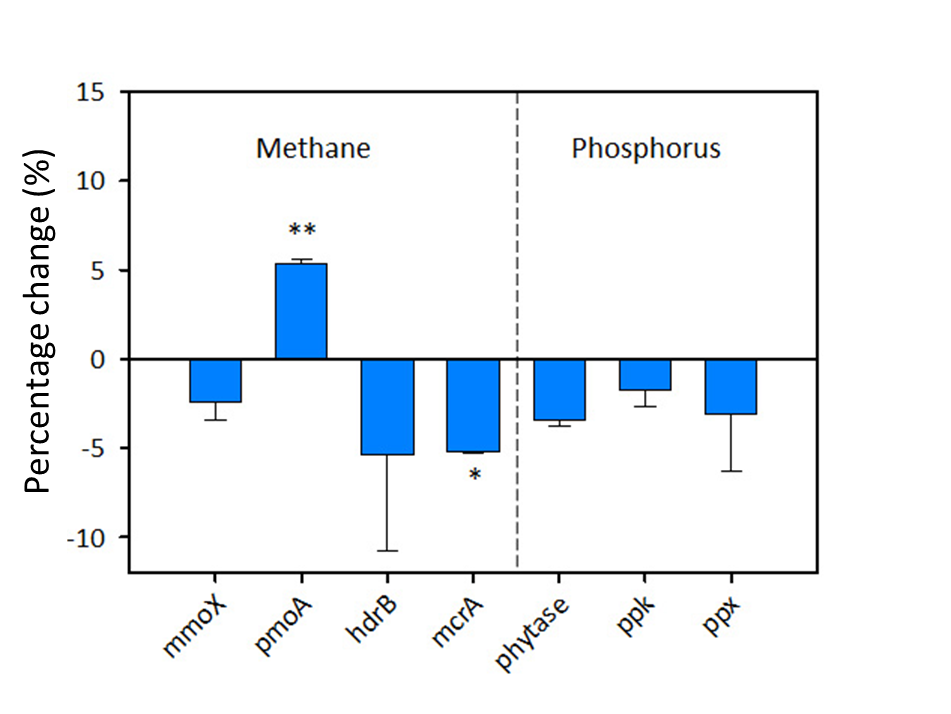


**Fig. S6** N deposition effects on *amoA* gene. The relative abundance of *amoA* is presented as the signal intensity difference between control and N deposited samples. Error bars represent standard errors. Blue bars represent genes derived from archaea (AOA), and pink bars represent genes derived from bacteria (AOB). Asterisks indicate significant differences. *, *P* < 0.050; **, *P* < 0.010.


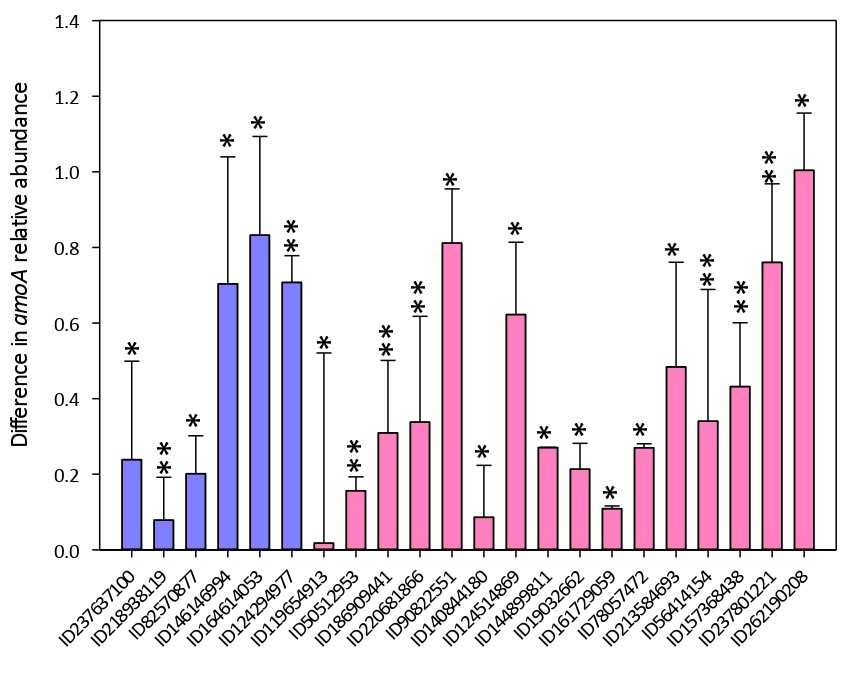

Supplement: Supplementary file 2 — Additional file 1: Table S1. Comparison of taxonomic and functional β-diversity between and within treatments. Table S2. Effects of N deposition on microbial taxonomic and functional diversity, as assessed by Shannon index. Table S3. Significantly changed representative OTUs calculated by difference analyses. Table S4. Topological properties of microbial functional gene networks. Table S5. Summary of soil and vegetation attributes in control and N deposited samples. Table S6. Mantel tests for correlations between a range of environmental attributes and quantitative measures of microbial community dissimilarity. Fig. S1. Comparison of the percentage change by N deposition for (a) microbial phyla; (b) N cycling genes; and (c) C cycling genes between using 32 and 4 samples as biological replicates. Fig. S2. The percentage change in relative abundances of microbial class induced by long-term N deposition. Asterisks indicate significant differences. *, P < 0.050; **, P < 0.010. Fig. S3. The percentage change in the relative abundance of major microbial genera induced by long-term N deposition treatment. All selected genera are significantly changed by N deposition treatment as calculated by the response ratio analysis. Fig. S4. The percentage change in the relative abundance of genes associated with C fixation induced by N deposition, calculated as 100*(( mean value in N deposited samples/mean value in control samples) – 1). Mean values and standard deviations are presented. Asterisks indicate significant differences. *, P < 0.050; **, P < 0.010. The numbers in the figure represent the pathways of C fixation. (i) 3-hydroxypropionate bicycle, (ii) Bacterial microcompartments, (iii) Calvin cycle, and (iv) Reductive tricarboxylic acid cycle. Fig. S5. The percentage change in the relative abundance of genes associated with methane and phosphorus cycling genes induced by N deposition, calculated as 100*((mean value in N deposited samples/mean value in control samples) – 1). M [file 40168_2022_1309_MOESM1_ESM.docx]
